# Systematic transcriptomic analysis of childhood medulloblastoma identifies N6-methyladenosine-dependent lncRNA signatures associated with molecular subtype, immune cell infiltration, and prognosis

**DOI:** 10.1186/s40478-024-01848-2

**Published:** 2024-08-28

**Authors:** Kandarp Joshi, Menglang Yuan, Keisuke Katsushima, Olivier Saulnier, Animesh Ray, Ernest Amankwah, Stacie Stapleton, George Jallo, Michael D. Taylor, Charles G. Eberhart, Ranjan J. Perera

**Affiliations:** 1 Department of Oncology, Sidney Kimmel Comprehensive Cancer Center, School of Medicine, Johns Hopkins University, 1650 Orleans St, Baltimore, MD 21231 USA; 2 Johns Hopkins All Children’s Hospital, 600 5th St. South, St. Petersburg, FL 33701 USA; 3 Genomics and Development of Childhood Cancers, Institut Curie, PSL University, Paris, 75005 France; 4 Cancer Heterogeneity Instability and Plasticity, Inserm U830, Institut Curie, PSL University, Paris, 75005 France; 5 SIREDO: Care, Innovation and Research for Children, Adolescents and Young Adults with Cancer, Institut Curie, Paris, 75005 France; 6 Keck Graduate Institute, Claremont, CA USA; 7 Medical College of Wisconsin, 8701 W Watertown Plank Rd, Milwaukee, WI 53226 USA; 8 Hematology-Oncology Section, Texas Children’s Cancer Center, Houston, TX 77004 USA; 9 Department of Pediatrics – Hematology/Oncology and Neurosurgery, Baylor College of Medicine, Houston, TX 77004 USA; 10 Department of Pathology, Johns Hopkins University School of Medicine, 720 Rutland Ave, Ross Bldg 558, Baltimore, MD 21205 USA

**Keywords:** Epitranscriptomics, Immunity, Long noncoding RNA, Medulloblastoma, N6-methyladenosine, Prognosis

## Abstract

Medulloblastoma, the most common malignant pediatric brain tumor, is classified into four main molecular subgroups, but group 3 and group 4 tumors are difficult to subclassify and have a poor prognosis. Rapid point-of-care diagnostic and prognostic assays are needed to improve medulloblastoma risk stratification and management. N6-methyladenosine (m6A) is a common RNA modification and long non-coding RNAs (lncRNAs) play a central role in tumor progression, but their impact on gene expression and associated clinical outcomes in medulloblastoma are unknown. Here we analyzed 469 medulloblastoma tumor transcriptomes to identify lncRNAs co-expressed with m6A regulators. Using LASSO-Cox analysis, we identified a five-gene m6A-associated lncRNA signature (M6LSig) significantly associated with overall survival, which was combined in a prognostic clinical nomogram. Using expression of the 67 m6A-associated lncRNAs, a subgroup classification model was generated using the XGBoost machine learning algorithm, which had a classification accuracy > 90%, including for group 3 and 4 samples. All M6LSig genes were significantly correlated with at least one immune cell type abundance in the tumor microenvironment, and the risk score was positively correlated with CD4^+^ naïve T cell abundance and negatively correlated with follicular helper T cells and eosinophils. Knockdown of key m6A writer genes *METTL3* and *METTL14* in a group 3 medulloblastoma cell line (D425-Med) decreased cell proliferation and upregulated many M6LSig genes identified in our *in silico* analysis, suggesting that the signature genes are functional in medulloblastoma. This study highlights a crucial role for m6A-dependent lncRNAs in medulloblastoma prognosis and immune responses and provides the foundation for practical clinical tools that can be rapidly deployed in clinical settings.

**Supplementary Information:**

The online version contains supplementary material available at 10.1186/s40478-024-01848-2.

## Introduction

Medulloblastoma (MB) is a tumor of the developing cerebellum usually seen in children. MB is broadly divided into four main molecular subgroups: WNT signaling-activated (WNT), sonic hedgehog signaling-activated (SHH) and non-WNT/non-SHH tumors split into two main subtypes: group 3 (Grp3), and group 4 (Grp4) [1, 2], although other subtypes have been identified [3]. Tumors in the WNT subgroup primarily harbor mutations in WNT signaling pathway genes such as *CTNNB1*, while SHH subgroup tumors harbor mutations in the SHH pathway such as *PTCH1*, *SUFU*, and *SMO* [4]. However, a lack of distinguishing mutations or copy number alterations (CNA) between Grp3 and Grp4 MB make them difficult to separate and manage. Recent attempts at subgroup classification have attempted to use epigenetic markers such as DNA methylation [4–6], while gene expression-based subgroup classification is challenging, as Grp3/Grp4 tumors exist as a continuum rather than as distinct subgroups [5]. While WNT MB is curable and certain non-*TP53* mutant subtypes of SHH patients also have a good prognosis, Grp3 and Grp4 tumors tend to be highly aggressive and progress to relapse or metastasize [6]. Recent single-cell RNA sequencing (scRNA-seq) studies have revealed distinct cells of origin for Grp3 and Grp4 tumors [7, 8]. Furthermore, treatment of patients with high-risk Grp3 MB with chemoradiotherapy and carboplatin improves the 5-year event-free survival by 19% [9]. Therefore, Grp3 and Grp4 subclassification according to cell type and molecular mechanism is vital for risk stratification, patient care, and improving management and outcomes. However, there is currently no cost-effective nor rapid clinical classification method.

Long non-coding RNAs (lncRNA) and circular RNAs (circRNA) are regulatory non-coding RNAs (ncRNAs) that play important roles in MB gene regulation and tumor progression [10–12]. Several lncRNAs and circRNAs have been reported to have oncogenic roles and act as potential targets for therapy [13–17]. However, this field is still in its infancy, with many new candidates still being actively researched and discovered. Adding to the complexity of RNA regulation and function, chemical modifications of RNA bases such as with N6-methyladenosine (m6A), pseudo-uridine, and inosine are prevalent RNA modifications that add a layer of regulatory information on RNA. These modifications occur co-transcriptionally and contribute to many aspects of the RNA life cycle including stability, translation, and transport. m6A dynamics is tightly regulated by group of methyltransferase proteins (‘writers’) and demethylases (‘erasers’), with another group of proteins able to recognize modified RNA (‘readers’) to exert specific functions [18, 19]. These group of writers, erasers, and readers can act as m6A regulator genes that play important roles in m6A dynamics and, in turn, gene regulation [20]. Modifications of ncRNAs, including lncRNAs, are notable, as they directly affect RNA stability and regulation, but the role of m6A in pediatric cancers including MB has not been explored in detail [21]. Existing data suggest that m6A has prognostic value and affects immune cell infiltration in brain cancers [22–27], and a well-characterized lncRNA *GAS5* has been shown to have an m6A-dependent role in tumor suppression in colorectal cancer (CRC). Methylated *GAS5* transcripts are degraded by the m6A reader YTHDF3, which increases expression of YAP and in turn drives tumor progression [28]. Cui et al. showed that downregulation of m6A writers *METTL3/METTL14* or m6A erasers *ALKBH5/FTO* reduces m6A on oncogenic *ADAM19* mRNA, increasing its expression and driving self-renewal of glioblastoma stem cell, leading to aggressive tumor behavior and metastasis [29]. Dong et al. showed that *ALKBH5* was responsible for m6A depletion on lncRNA *NEAT1* in glioblastoma cells and increased transcript stability, allowing the transcriptional repressor SFPQ to relocate from the *CXCL8* promotor to nuclear paraspeckle bodies, leading to upregulation of IL-8 in the tumor microenvironment and tumor-associated macrophage (TAM) recruitment. The immunosuppressive effect of TAMs allowed tumor progression and was partially rescued by IL-8 overexpression in ALKBH5-deficient GBM cells [30]. NEAT expression was associated with immune checkpoint blocker therapy in glioblastoma (GBM) patients and increased NEAT1 expression in M1 polarized macrophages led to secretion of inflammatory cytokines [31]. LncRNAs have previously been used to subclassify MB, but not with respect to m6A modifications [32]. We therefore reasoned that the diagnostic and prognostic accuracy of biomarkers for MB could be increased by integrating two different layers of regulatory information, lncRNAs and their m6A modification.

To achieve this, we analyzed bulk transcriptome data from over 400 MBs to identify m6A-associated lncRNA signatures. We constructed a co-expression network and identified modules showing representation of m6A regulators identified from a previous study [33]. Co-expressed lncRNAs in these modules associated with prognosis were selected as signature genes, which were used to calculate a risk score which, combined with other clinical parameters, predicted overall survival (OS) for risk stratification. Furthermore, using a machine learning model, the m6A-lncRNA signature was used for subgroup classification. Finally, we correlated the expression of each m6A-associated lncRNA and its signature-derived risk score with immune cell infiltration in the tumor microenvironment. Patient stratification into high and low risk groups based on risk score revealed significant differential expression of immune checkpoint and ligand genes. Collectively, we identify a new role for m6A-modified lncRNAs in immune-related functions and novel candidates for further functional characterization and testing as immune modulatory agents in MB.

## Methods

### Data collection and pre-processing

Bulk transcriptome data and associated metadata for three discovery cohorts of MB patients were analyzed. Raw RNA-seq counts were obtained from OpenPBTA (*n* = 119) [34] and Williamson et al. (*n* = 331) [5]. Alignment files in BAM format were retrieved from the St. Jude Cloud (*n* = 89) [35], with gene counts generated using *stringtie* [36]. Additionally, independent validation cohorts GSE164677 (*n* = 31) and the MAGIC cohort (*n* = 507) (unpublished) were used to validate the risk score predicting OS. Validation cohorts (GSE181293, GSE164677, and GSE85217; *n* = 839) were used for subgroup classification accuracy testing. A summary of the discovery and validation cohorts can be found in Supplementary Table 1. Samples with missing age and sex metadata were excluded from the analysis, and genes represented in all three cohorts were selected for analysis. Data were normalized using the *vst* function in DESeq2 [37], and subgroup clustering of the samples was visualized using a principal component analysis (PCA) plot. In order to perform cross-platform data integration and validation of the subgroup classification signature in RNA-seq and microarray-derived datasets, *ComBat* batch correction from the *sva* package [38] was performed on normalized gene expression profiles of discovery and validation cohorts.

### Construction of co-expression networks and m6A-associated lncRNA gene selection

A co-expression network was constructed using the weighted gene co-expression network analysis (WGCNA) package in R [39]. An appropriate soft threshold was identified using power analysis for network construction. The constructed unsigned co-expression network contained a minimum module size of 50 genes and a maximum block size of 20,000. m6A-associated modules were identified based on representation of at least one out of 22 m6A regulatory genes within the module. Soft threshold selection to identify the power for network construction was performed with *pickSoftThreshold*, and an unsigned network of genes was constructed using *blockwiseModules.* LncRNA genes co-expressed with m6A regulator genes within m6A-associated modules were defined as m6A-associated lncRNA genes.

### Survival analysis and gene selection for calculating the M6LSig risk score

LASSO-Cox regression-based feature selection was performed using the *glmnet* package to identify m6A-associated lncRNA genes associated with OS based on gene expression using the *rms* package in R, with age and sex as covariates. We selected genes with *P*-values < 0.05 as m6A-associated lncRNA signature (M6LSig) genes significantly associated with OS. A patient’s risk score [M6LSig risk_score] was calculated based on M6LSig expression using the following formula:

1 $$\:\:M6LSig\:risk\_score={\sum\limits_{k=1}^{n} }coef\:\left({x}_{k}\right)*{x}_{k}$$

where $\:{x}_{k}$ is the expression of the k^th^ gene, and $\:coef\:\left({x}_{k}\right)$ is the coefficient of the Cox regression of the k^th^ gene.

### M6LSig risk score-based prognostic model for medulloblastoma

Risk scores were calculated for all samples, and the median risk score was used to divide the samples into low- and high-risk groups based on lower and higher risk scores, respectively. Associations between risk score, age, and sex and OS were evaluated using a Cox proportional hazards (cph) model in the *rms* R package. Using the cph model generated using risk scores, age, and sex as variables and OS time and status, a points-based nomogram was developed to assess OS. Nomogram accuracy was assessed with calibration curves and receiver operating characteristic (ROC) curves in independent validation cohorts.

### Machine learning model to classify medulloblastoma samples by lncRNA gene expression

*Boruta* package was used to identify the minimum set of genes capable of accurately subgrouping MBs. Briefly, the Boruta training function was run for 100 iterations to identify the gene set required for subgroup classification based on gene expression of lncRNAs co-expressed with m6A regulator genes in WGCNA modules.

Samples from the discovery cohort were divided into training and test sets in an 80:20 ratio. The training set was used to generate a model for subgroup classification. Eight algorithms were generated using the *caret* package in R for subgroup prediction based on M6LSig gene expression: glmnet, linear discriminate analysis (LDA), support vector machine (SVM), XGBoost, gradient boosting (GB), random forest (RF), K-nearest neighbors (KNN), and C50. We used the test set to evaluate model performance, precision, recall, and F1 scores, and multiclass ROC curves were calculated to assess the classification accuracy of each model. The performance of the top 5 models was evaluated using multi-class ROC curves using independent validation set not used for training the classifier models. The best performing model was chosen based on the highest multi-class accuracy.

### Functional characterization of differentially expressed genes between low- and high-risk groups

Significant differentially-expressed genes (DEGs; adjusted *P*-value < 0.05) were identified between high- and low-risk groups using *DESeq2* in R. We further ranked the expressing genes based on their test statistics for gene ontology and KEGG pathway gene set enrichment analysis (GSEA) using the *clusterprofiler* package. The significance of differential expression of 19 immune checkpoint-related genes and ligands was also examined.

### Association between M6LSig and immune cell infiltration in the tumor microenvironment

**Fig. 1:**
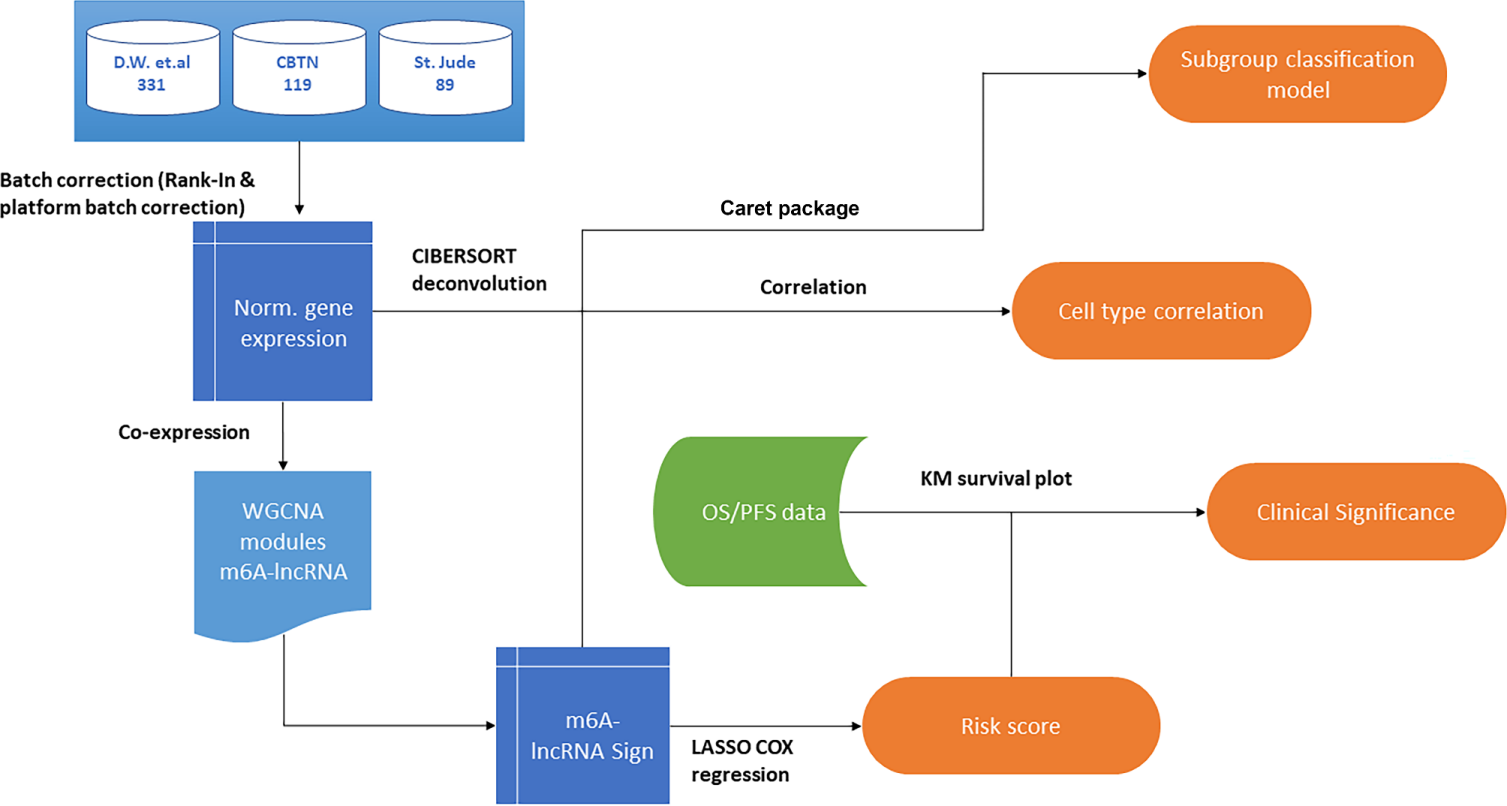
Workflow depicting the datasets analyzed and the steps involved in identifying m6A-associated lncRNA expression-based risk scores for predicting the survival of medulloblastoma patients

Immune cell infiltration in the tumor microenvironment (TME) was identified using the cell type deconvolution method CIBERSORTx [40], with the LM22 dataset of 22 immune cell type gene expression patterns used as a reference set. Normalized gene expression, transcripts per million (TPM) for samples in the discovery cohort and LM22 reference datasets were used as references for immune cell deconvolution. Significant cell type proportions (*P* < 0.05) were selected, and correlations between individual M6LSig gene expression and risk scores with immune cell type proportions were calculated. Using R, the correlations between individual gene expression to cell type proportions and risk score were visualized. The complete analysis workflow is shown in Fig. 1.

### Cell lines and cell culture

The human MB cell line D425-Med was purchased from Sigma-Aldrich (St. Louis, MO), which was STR profiled and *Mycoplasma* tested. D425 cells were cultured in DMEM/F12 with 10% FBS and 1% penicillin/streptomycin. Cells were grown in a humidified incubator at 37 °C in 5% CO_2_, and the culture medium was replaced every 3 to 4 days. The cells were gently trypsinized (0.05%, Gibco, Thermo Fisher Scientific, Waltham, MA) for subculture.

### Quantitative real-time PCR

Total RNA was purified as per the manufacturer’s instructions using the Direct-zol RNA Miniprep kit (Zymo Research, Irvine, CA). RNA yields were measured with a NanoDrop 8000 spectrophotometer (Thermo Fisher Scientific). High-Capacity cDNA Reverse Transcription Kits (Applied Biosystems, Waltham, MA) were used to reverse transcribe 500 ng RNA, with quantitative PCR performed using SYBR Green Master Mix assays (Applied Biosystems). The primer sequences are listed in Supplementary Table 2.

### Cell proliferation assay

D425 cells were harvested in the logarithmic phase and cultured in 96-well plates for varying times for the cell proliferation assay. 20 µl MTS solution (Promega, Madison, WI) was added to each well, and the absorbance (optical density value) was measured at 490 nm on the EnVision 2105 microplate reader (PerkinElmer, Waltham, MA) after 2 h.

### Statistical analysis

Risk score was calculated according to Eq. 1. Samples were divided into high and low risk group based on the risk score value being higher or lower than the median risk score value. Differential expression analysis between high and low risk group samples was performed using DESeq2. Adjusted *p*-value cut-off 0.05 was used to identify significant differentially expressed genes between high risk and low risk patient groups. Cell proliferation and qRT-PCR data is represented as mean ± SD. Significance of differences between the groups were calculated using standard student’s t-test. *P*-value cut-off of 0.05 was used for determining statistical significance.

## Results

### Identification of an m6A regulator and lncRNA gene co-expression network

**Fig. 2:**
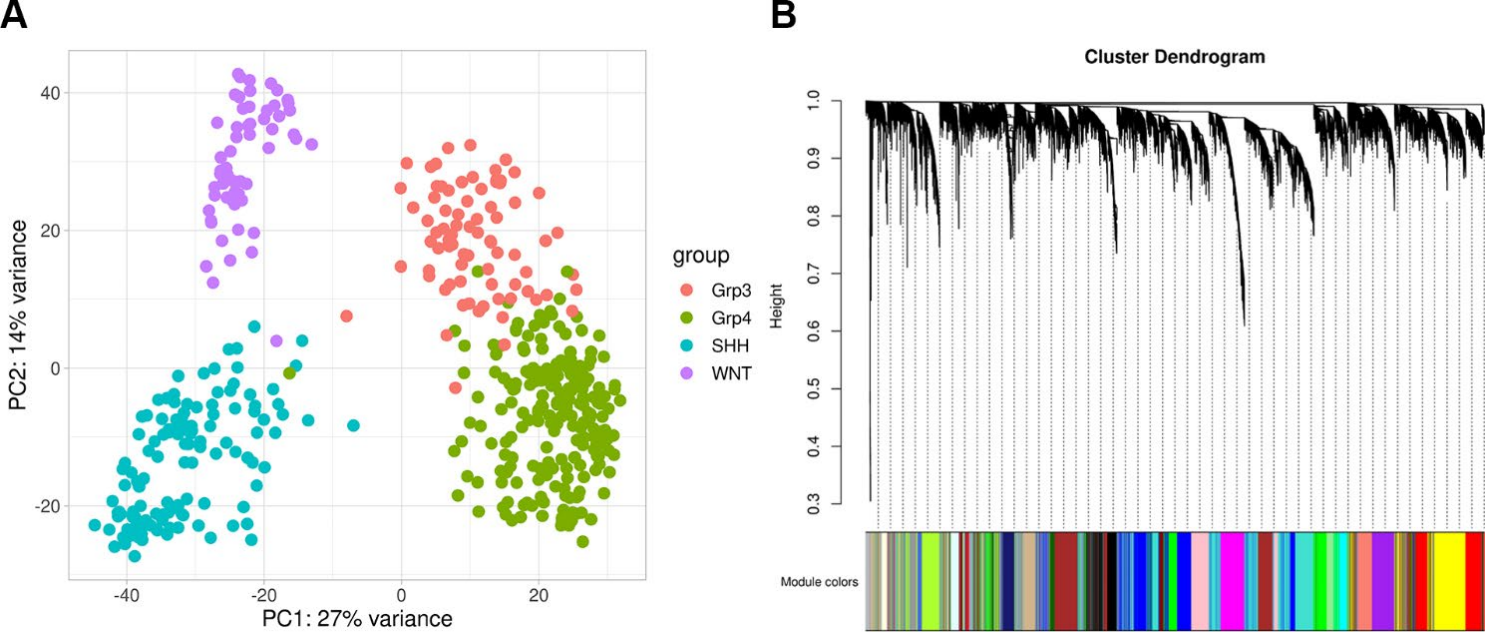
**(A)** PCA plot of samples clustering by subgroup after batch correction and cross platform normalization. **(B)** Dendrogram showing correlation of genes within gene co-expression modules identified by WGCNA. Genes co-expressed within the same module are represented by the same color

The discovery cohort consisted of 539 samples (OpenPBTA; Williamson et al. and St. Jude cohorts) and 18,872 genes. Samples with missing clinical metadata, such as age and sex, were excluded. The PCA plot shows sample clustering by subgroup for 469 samples from RNA-seq datasets after normalization (Fig. 2a). Gene expression profiles from 469 samples were used to build WGCNA co-expression modules. An unsigned co-expression gene network with 23 modules was constructed (Fig. 2b). Modules with at least one m6A regulator gene were identified as m6A-associated modules; 74 lncRNA genes (gencode v44) were significantly co-expressed with these m6A regulatory genes (Supplementary Table 3a-b).

### Construction of the m6A-lncRNA gene signature

**Fig. 3:**
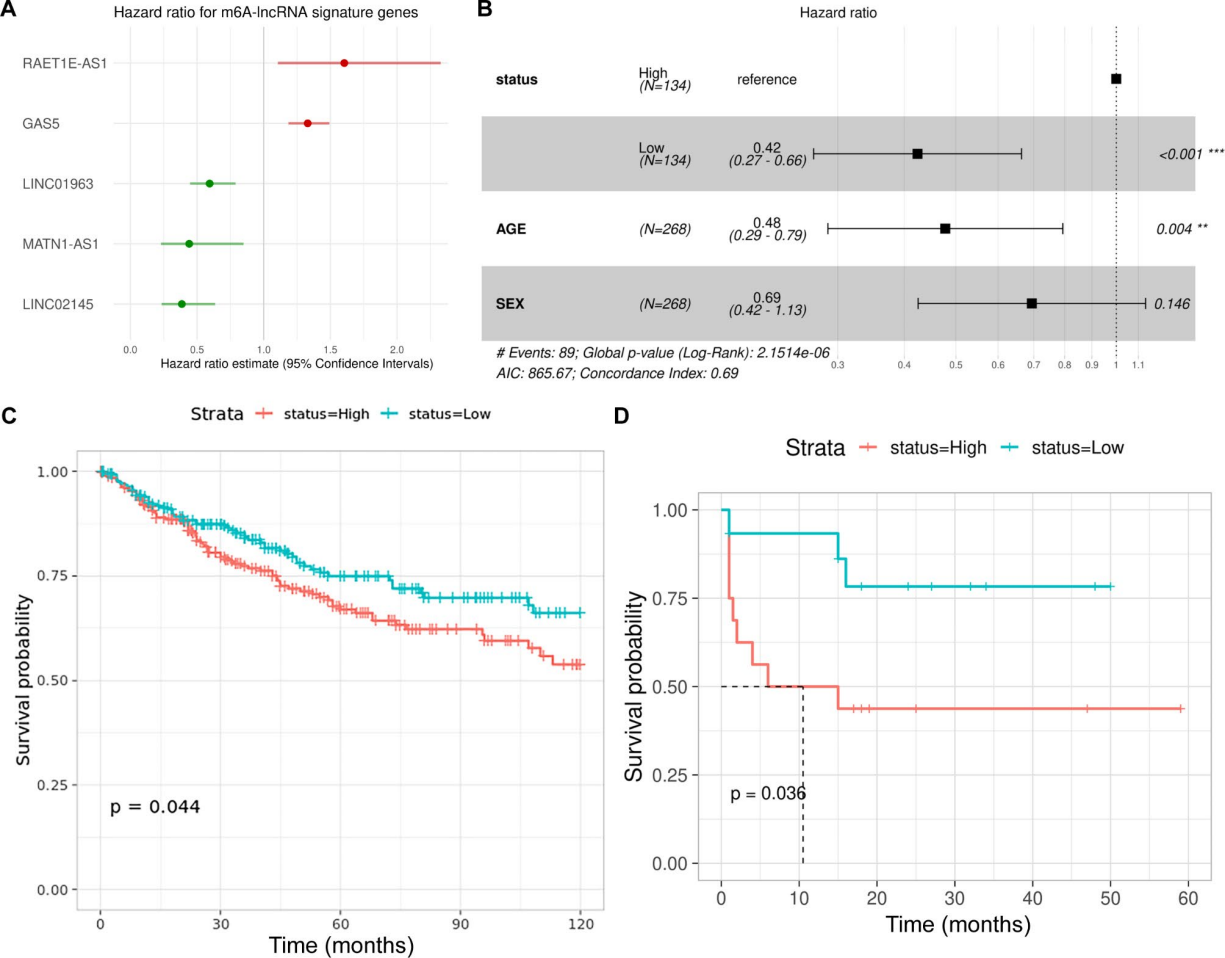
**(A)** Plot with hazards ratio on the X-axis for each of the five genes significantly associated with OS. **(B)** Forest plot showing OS association and hazards ratio for each co-variate including risk score. **(C-D)** K-M plots derived from risk scores in independent MB datasets to validate the five-gene signature-derived risk score’s association with OS.

m6A-associated lncRNA genes were associated with patient OS. In total, 268 samples had survival metadata available, including age and sex covariates. Survival time was censored at 10 years. LASSO-Cox regression analysis was performed to identify m6A-associated lncRNAs associated with survival by selecting an appropriate lambda from the regression analysis (Supplementary Fig. 1a-b), and five lncRNAs were significantly associated with OS (Fig. 3a). An m6A-lncRNA signature risk score was calculated for each patient based on the expression of these five lncRNAs, and samples were divided into those above (high risk) or below (low risk) the median of the risk score. Using Cox multivariable regression, we found significant associations between risk score and OS, with low-risk scores showing better survival (hazard ratio (HR) = 0.42; 95%CI 0.27–0.66; *P* < 0.001). Similarly, patient age (HR 0.48, 95%CI 0.29–0.79; *P* = 0.004) was associated with OS and, while sex had an HR of 0.69 (95%CI 0.42–1.13; *p* = 0.146) for OS, this was not quite significant (Fig. 3b). However, given the marginal significance and previous reports of a significant group 3 bias for male vs. female patients (2:1) and a significantly poorer five-year survival for males [41], we retained sex in subsequent prognosis calculations.

**Fig. 4:**
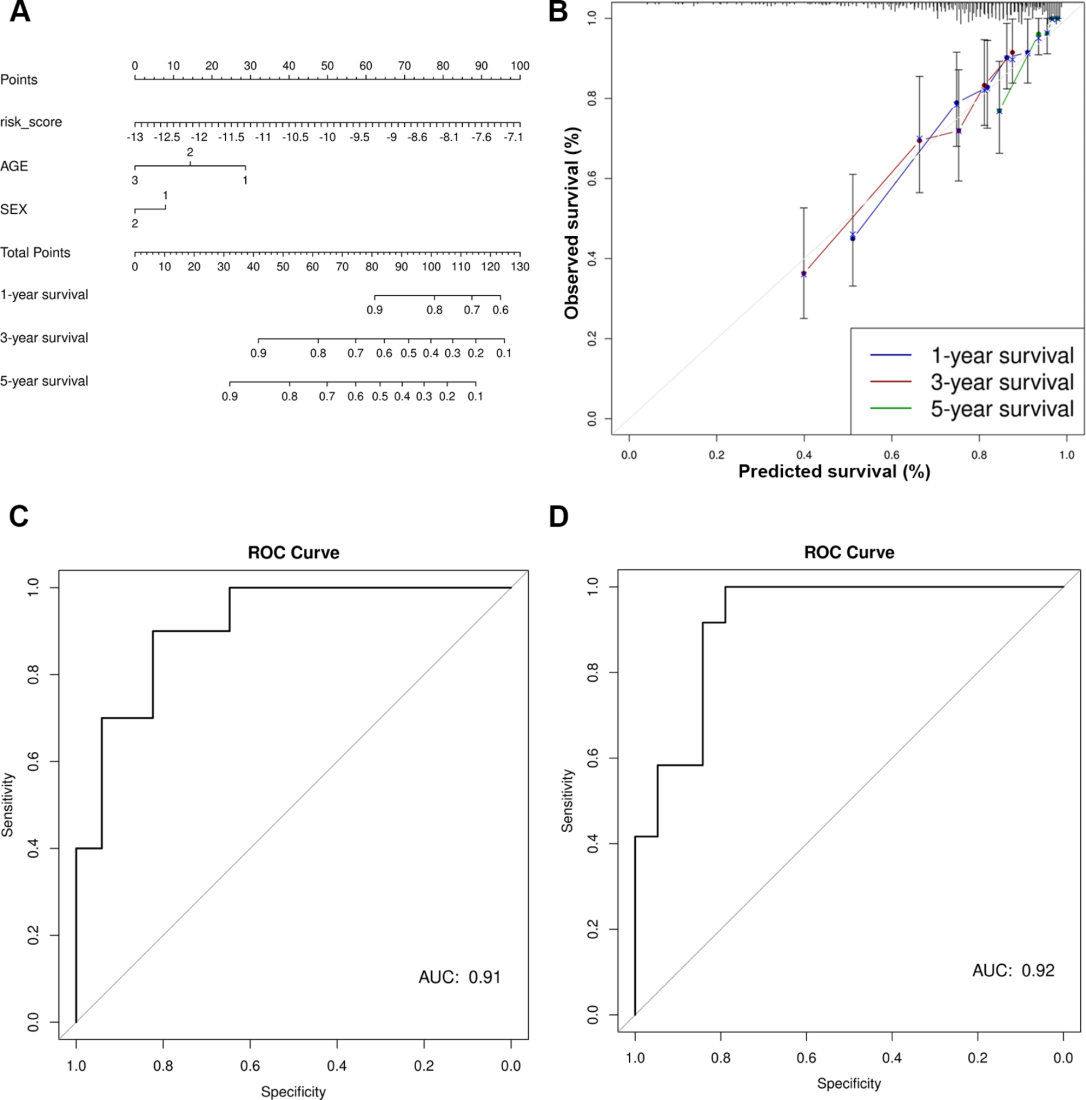
**(A)** Nomogram to predict 1-year, 3-year and 5-year OS in patients with MB based on risk score, age, and sex. **(B)** Calibration curves representing the accuracy of the survival prediction model based on risk score, age, and sex of the patients. **(C-D)** ROC curves showing the predictive accuracy of the nomogram-based survival probability of patients with MB.

Discovery datasets were divided into training and test sets (90:10, respectively). Cox proportional hazards was used to model the training data and calculate the probability of 1-, 3-, and 5-year survival based on five m6A-lncRNA signature risk scores, age, and sex. The model was used to construct a nomogram (Fig. 4a), which was evaluated using calibration curves (Fig. 4b). Calibration curves provide information about agreement between observed versus expected probabilities of the estimates, where a linear relationship with a slope of the curve close to 1 indicates a high level of accuracy in predicting the OS of patients with MB. The best survival predictions were obtained using risk score, age, and sex for 1- and 3-year survival. Independent RNA-seq datasets (GSE164677 and MAGIC cohort) were used to validate the association of the five gene signature-derived risk score’s association with OS (Fig. 3c-d). Test data were used to generate ROC plots, and an area under curve (AUC) > 0.9 indicated a high accuracy of the five gene signature derived risk score in predicting OS (Fig. 4c-d).

### Machine learning models for subgroup classification based on m6A-associated lncRNA gene expression profiles

**Fig. 5:**
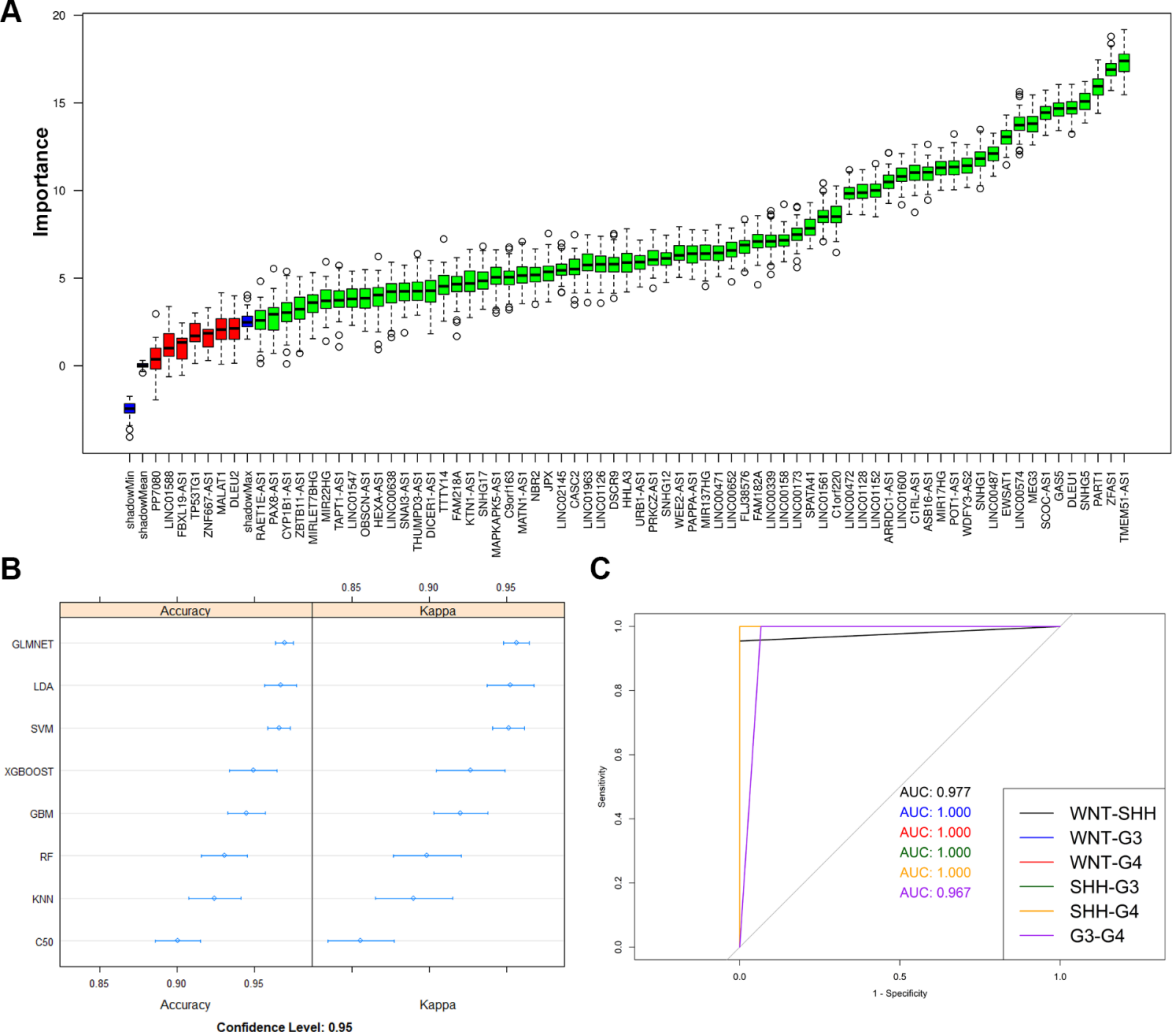
**(A)** Feature importance plot showing importance of each gene in subgroup classification. Genes in green are confirmed important genes and genes in red are rejected genes. **(B)** Evaluation of model accuracy of the eight ML models used for subgroup classification based on the 67 m6A-lncRNA gene signatures. **(C)** AUC plots show the XGBoost model-based classification accuracy between individual MB subgroups in test cohorts

Normalized and batch-corrected gene expression from the discovery cohorts (*n* = 469) were next used to train machine learning-based subgroup classification models. Out of 74 lncRNAs, 67 were identified as important features for subgroup classification using *Boruta*-based feature selection. Samples were randomly divided into training and test sets (80:20 ratio, respectively), and eight ML methods were assessed for accuracy in subgroup classification (Fig. 5a). LDA was the best-performing ML method, with a median accuracy of 97.3% (Table 1). Using test and independent validation cohorts (GSE164677, GSE181293, GSE164677, and GSE85217), the multiclass AUCs for the top five performing models were calculated, together with overall subgroup classification accuracy, per-group classification accuracy, and F-score. Table 2 shows the F-score for each model for individual subgroup classification accuracy and overall accuracy. Glmnet and XGBoost were the two best classifiers. XGBoost performed slightly better for Grp3 classification (Fig. 5b and Supplementary Fig. 2) and also performed the best for Grp3 vs. Grp 4 classification (Fig. 5b).

**Table 1:** Accuracy of eight machine learning models for subgroup classification based on the expression of 67 m6A-associated lncRNA genes

ML model	Min.	1st Qu.	Median	Mean	3rd Qu.	Max.
RF	0.87	0.91	0.93	0.93	0.95	0.97
GBM	0.91	0.93	0.95	0.95	0.96	0.97
C50	0.84	0.89	0.89	0.90	0.92	0.95
LDA	0.93	0.95	0.97	0.97	0.98	0.99
GLMNET	0.96	0.96	0.97	0.97	0.97	0.99
KNN	0.88	0.89	0.93	0.92	0.95	0.97
SVM	0.95	0.96	0.97	0.97	0.97	0.99
XGBOOST	0.91	0.93	0.95	0.95	0.97	0.99

**Table 2:** Test set-based accuracy, F score, and AUC of the top five ML classifiers for subgroup classification of samples based on the expression of 67 m6A-lncRNA genes

	Discovery cohort					Validation cohort				
	Class: WNT	Class: SHH	Class: Grp3	Class: Grp4	Multi-class AUC	Class: WNT	Class: SHH	Class: Grp3	Class: Grp4	Multi-class AUC
GLMNET	0.96	1	0.9333	0.988	0.97	0.82873	0.9255	0.7959	0.9206	0.88
SVM	0.96	0.9796	0.9655	0.988	0.988	0.82222	0.9276	0.8	0.9231	0.87
LDA	1	1	1	1	1	0.8306	0.9038	0.7744	0.885	0.86
XGBOOST	0.96	0.9583	0.8571	0.9647	0.955	0.82418	0.9155	0.8127	0.9148	0.88
GBM	0.96	0.9362	0.8387	0.9639	0.955	0.83243	0.9026	0.787	0.8976	0.87

**Table 3:** Correlation and significance (*P*-values) of risk scores, with each cell type identified by gene expression-based cell type deconvolution

Cell type	*R*	*P*-value
naïve T cells CD4	0.14	0.02
Follicular helper T-cells	-0.13	0.04
Eosinophils	-0.13	0.04

### Functional and immune characterization of the m6A-assocaited lncRNA signature and risk score

**Fig. 6:**
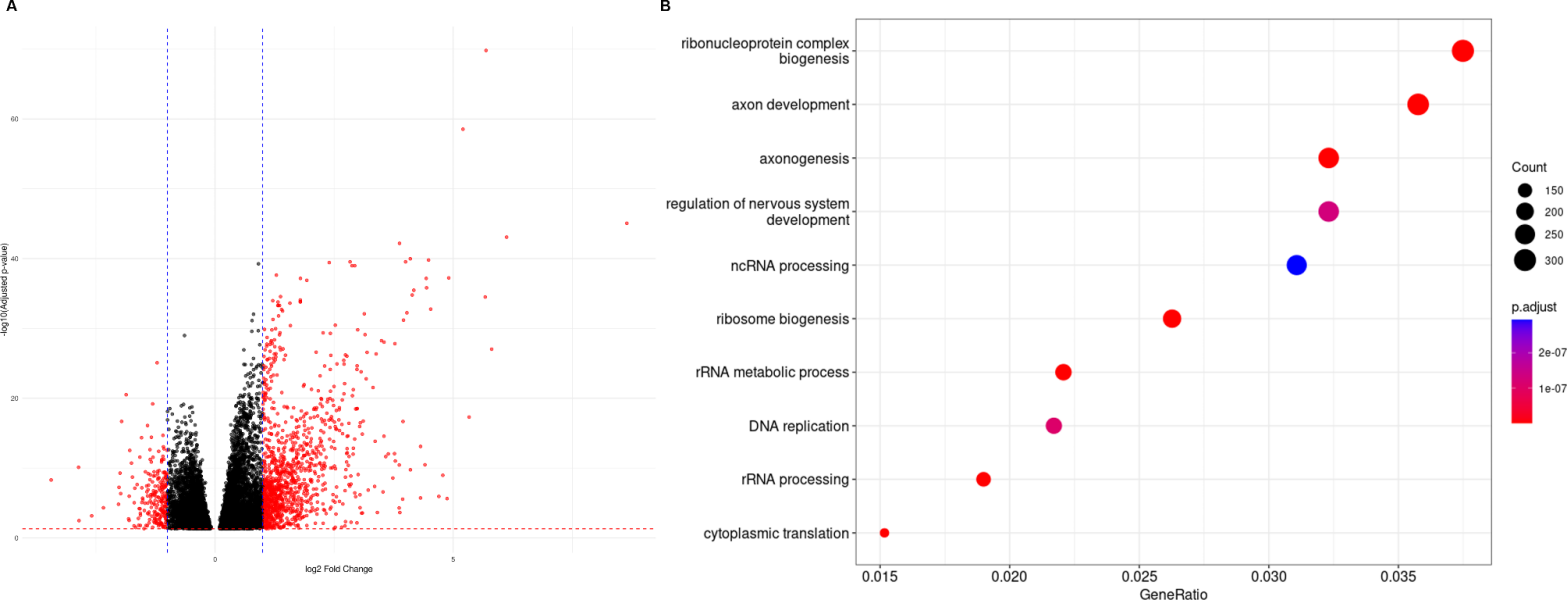
**(A)** Volcano plot of DEGs between high- and low-risk patients. Each dot represents a gene, and dots colored in blue are significantly differentially expressed (adj.P.val < 0.05). **(B)** Gene ontology biological processes enriched for differentially expressed genes between high and low risk patient groups

**Fig. 7:**
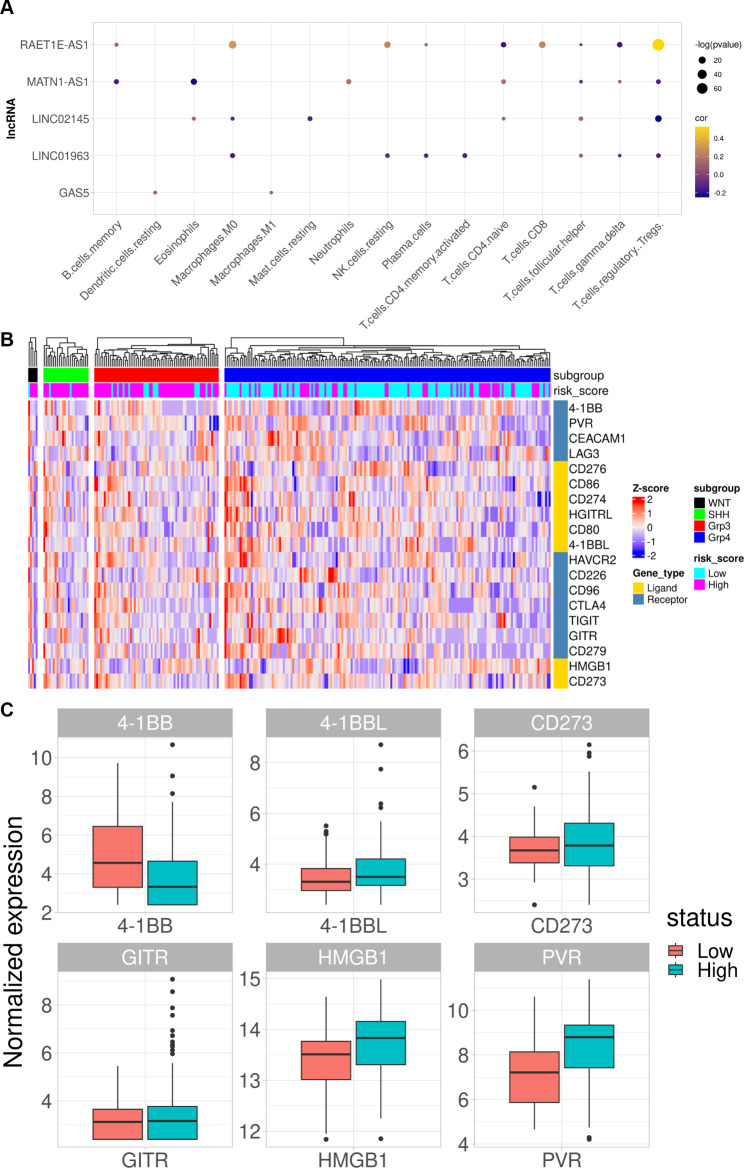
**(A)** Dot plot showing correlations (dot color) and significance (dot size) of lncRNA gene expression with cell type abundance calculated by CIBERSORTx. **(B)** Heatmap showing normalized expression of 19 immune checkpoint and ligand genes. Top row shows patient subgroups, and the panel below shows the risk group of the patient. The receptor or ligand label of the gene is represented in the vertical column with L & R labels. **(C)** Significant differences in expression of six IC ligands and receptors in high- and low-risk patients

Differential expression analysis of genes between high- and low-risk patient groups identified 10,676 DEGs, represented as a volcano plot in Fig. 6a. Functional characterization of DEGs identified key biological processes involved in ribonucleoprotein complex biogenesis, axon development and axonogenesis, and regulation of nervous system development, amongst others (Fig. 6b). Immune cell proportions within the TME were next calculated by gene expression-based cell type deconvolution using CIBERSORTx and the LM22 dataset, which represents the expression of 547 genes in 22 immune cell types derived from scRNA-seq data. Cell type proportions per sample were calculated for 499 samples profiled by RNA-seq, which were then correlated with the expression of individual genes in the m6A-lncRNA signature and the risk score. All lncRNAs significantly correlated with at least one immune cell type in the TME, as determined by cell type deconvolution analysis (Fig. 7a). The risk score was found to be positively correlated with abundance of naïve T cells CD4 and negatively correlated with follicular helper T-cells and eosinophils in tumor microenvironment (Table 3). The differential expression of 19 immune checkpoint (IC) and ligand genes were also examined in high- and low-risk patients (Fig. 7b), and six IC genes were significantly differentially expressed (Fig. 7c).

### *METTL3* and *METTL14* knockdown affects cell proliferation of Grp3 MB cells and expression of M6LSig genes

**Fig. 8:**
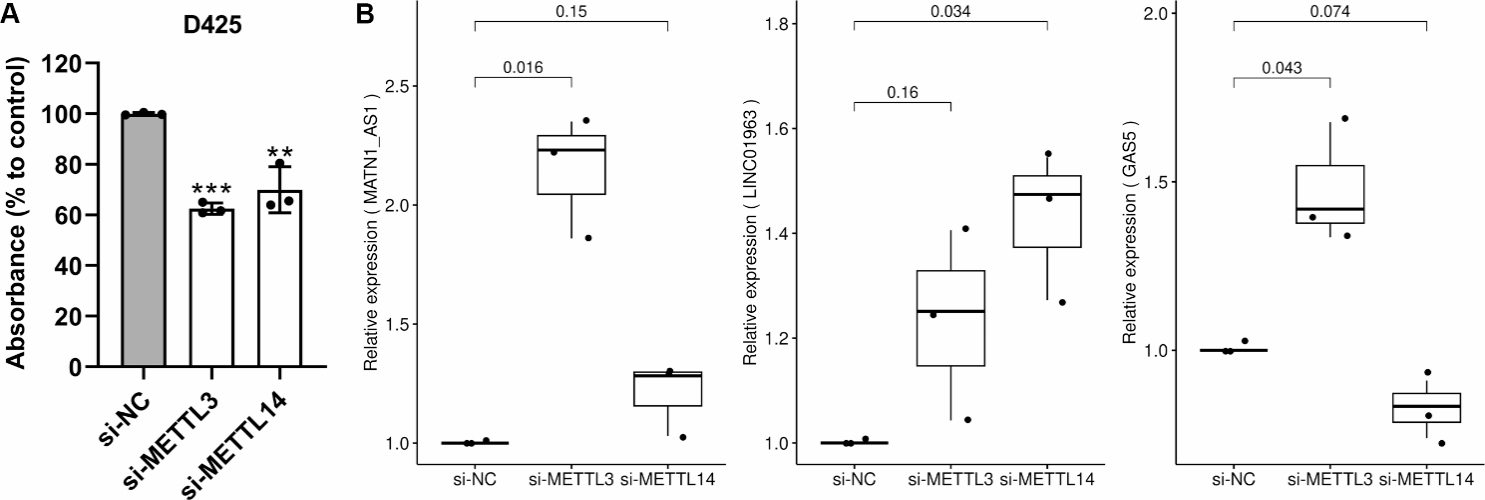
**(A)** Cell proliferation assay showing decreased proliferation of D425 Grp3 MB cells upon knockdown of m6A writers *METTL3* and *METTL14*. **(B)** qRT-PCR validation of dysregulation of three M6LSig genes upon knockdown of *METTL3* or *METTL14* in D425 cells compared with control samples

Finally, we knocked down *METTL3* and *METTL14*, key m6A writers, using siRNAs in D425 Grp3 MB cells. There was an average 62.5% and 70% reduction in proliferation of D425 cells after 72 h of *METTL3* and *METTL14* knockdown (Fig. 8a). qRT-PCR of the five genes in the signature showed that three lncRNAs (*MATN1-AS1*, *LINC01963*, and *GAS5*) were significantly upregulated in at least one of the knockdown condition samples (si*METTL3* or si*METTL14*) compared with control (siNC) (Fig. 8b). *RAET1E-AS1* and *LINC02145* were not expressed in this cell line, as confirmed in RNA-seq data of D425 cells in the CCMA database [42] (expression 0 or < 1 CPM).

## Discussion

Medulloblastoma (MB) is one of the most prevalent childhood brain tumors, with the highest incidence in children aged 10–14 years [43]. Children aged under three years have a higher risk of death from the disease, especially those with Grp3 or Grp4 tumors [6]. The recurrence rate for MB is high at 30% [44]. Different MB subgroups have different etiologies and cells of origin [4], and the resulting tumors progress differently, requiring personalized management. Subgroup classification requires histopathological analysis, but the Grp3 and Grp4 subgroups are challenging to differentiate, with costly and time-consuming DNA methylation-based classification currently the gold standard for Grp3 and Grp4 classification. There is therefore a clinical need for rapid point-of-care diagnostics for accurate subgroup classification and prognostication, especially in low-resource settings.

Our group previously reported a significant role for ncRNAs in MB subgroup classification and therapy [15–17, 32], and previous studies have reported the dynamics and dysregulation of m6A regulators in MB patients [21, 45]. RNA methylation is vital for regulating lncRNA-related disease progression and is an important therapeutic target. Nevertheless, m6A-based lncRNA regulation of tumor progression and clinical outcomes in MB have largely remained unexplored.

Here we systematically analyzed bulk transcriptomes from MB patients and integrated clinical traits and metadata to identify an m6A-associated lncRNA gene signature (M6LSig) with diagnostic and prognostic potential. LncRNAs co-expressed with m6A regulators are potentially regulated in an m6A-dependent manner. Our analysis revealed a five-gene signature significantly associated with prognosis in the context of age and sex. Due to intragroup heterogeneity and significantly different prognostic outcomes of subtypes within each subgroup, additional covariate of subgroup classification was not included in the model. The risk score calculated based on the expression values of these five genes was significantly associated with OS. High concordance of observed versus expected probabilities on calibration curves indicated that the model generated for predicting OS was highly accurate for one-, three-, and five-year survival. To aid clinical implementation, we generated a nomogram requiring the patient’s risk score, age, and sex for OS prediction. Eight ML-based classification models were also evaluated to determine the most precise algorithm for subgroup classification based on the 67-genes signature. XGBoost outperformed the other approaches, with > 90% accuracy for subgroup classification. These models have direct clinical application and are adaptable to diverse gene analysis platforms. Compared to other high throughput techniques dependent on quantifying bulk transcriptomes or DNA methylation profiles from patients, our small panel of genes are easier to scale and implement in clinical settings, even with limited resources availability. Although the roles of the lncRNAs forming this signature are not currently understood in MB, they have been implicated in many other cancers. For example, GAS5 has been reported to have prognostic value in CRC, where GAS5 regulates the transport and decay of YAP, an oncogene responsible for tumor progression in the disease. YTHDF3 can recognize and degrade m6A-containing GAS5 transcripts and hence negatively impact YAP degradation [28]. Further work is now needed to explore the functions and regulation of these lncRNAs.

Immune cell infiltration in the TME significantly impacts tumor progression, patient survival, therapy responses, and metastasis, including in MB, where CD8^+^ T cell infiltrates are associated with prognosis of MB patients. However, most MB tumors are considered ‘cold tumors’, i.e., the immune response is suppressed or inactive. Hence, immune modulation is a potentially important strategy for treating MB tumors, and novel targets for immune modulation hold important therapeutic and commercial potential. We explored correlations between expression of the five m6A-associated lncRNA genes and the abundance of 22 immune cell types within the MB TME, and all five genes showed significant positive or negative correlations with at least one of the 15 immune cell types (Fig. 6A). Correlating risk scores with immune cell type abundance in the TME identified a negative correlation with follicular helper T cells and eosinophils and a positive correlation with naive CD4^+^ T cells. Hence, the role of m6A-associated lncRNAs in regulating T cell and eosinophil infiltration and disease outcome is a novel therapeutic avenue for further exploration.

Clinically-applicable risk scores are important for personalized medicine. The result shown in Fig. 7b reports the dynamic expression patterns of the immune checkpoint and ligand genes in high- and low-risk patients, classified by their assigned subgroups. The plot shows the heterogeneity in the expression pattern of the immune checkpoint and ligand genes in the risk score-based stratified population of patients. Risk stratification by risk score is distinct from the subgroup classification-based risk stratification and that expression of immune checkpoint and ligand genes in samples with the same assigned subgroups are not similar. This indicates that an immune therapy approach and understanding the tumor microenvironment might involve additional risk factors than subgroup classification alone. Patients with a high-risk score showed significantly increased expression of 4-1BB, which is a positive stimulator of an anti-oncolytic immune response [46, 47]. Previous reports have shown positive outcomes for patients stimulated with anti-4-1BB antibodies, and combined low-dose radiation and anti-4-1BB antibodies have also been explored as a potential alternative therapy in MB patients [47, 48]. Taken together, our newly developed risk score has many clinical and personalized medicine applications in MB treatment and care.

The m6A writers *METTL3* and *METTL14* were significantly associated with proliferation of Grp3 cells in vitro. Zhang et al. similarly showed that *METTL3* knockdown in SHH MB cell lines (DAOY and ONS-76) significantly impacted cell proliferation [45]. Hence, m6A regulatory genes are important for tumor progression and growth. Our results also indicate that manipulation of part of the m6A machinery has a significant impact on the regulation of many genes including three of the five lncRNAs forming part of the M6LSig in Grp3 MB cells, suggesting that these lncRNAs may be functional. Taken together, these results highlight a key role for m6A regulators in MB tumor growth and potentially influencing expression of prognostic lncRNAs in an m6A-dependent manner.

The results of this study highlight a crucial role for m6A-dependent lncRNAs in MB prognosis and immune responses. Further exploration and functional characterization of these identified genes and pathways is now required to determine the role of m6A in regulating immune cell infiltration. Our nomogram provides the basis for a useful and practical tool that can be rapidly deployed in clinical settings for translational applications.

## Conclusions

We performed systematic in-silico analysis of publicly available bulk transcriptomes generated from the tumors of MB patients to identify a five gene m6A-associated lncRNA signature potentially linked to tumor progression and patient survival. Risk score derived from the five-gene signature has prognostic prediction ability. Another 67 gene lncRNA signature, which also contains the five prognostic lncRNAs, was identified with capability to perform subgroup classification with high accuracy. Our results indicate that m6A depletion by m6A writers (*METTL3* and *METTL14*) knockdown affected the expression of a subset of these signature lncRNA genes in Grp3 MB cell line. Depletion of global m6A from transcriptome by m6A writers (*METTL3* and *METTL14*) knockdown negatively affected the Grp3 MB cell line proliferation. Patients stratified by their risk score into high and low risk groups showed different gene expression patterns and biological processes. The expression of immune checkpoint genes and ligands was significantly different between high and low risk group patients. Expression of five lncRNA prognostic genes was also found to be significantly correlated with abundance of a subset of immune cells within tumor microenvironment. Taken together, these findings indicate the use of risk score-based stratification as potential marker for personalized immune therapy development application.

### Electronic supplementary material

Below is the link to the electronic supplementary material.

**Supplementary Table 1.** Patient demographic and clinical information of the discovery and validation cohorts used in this study

**Supplementary Table 2.** Primer sequences used for qRT-PCR validation of five lncRNA signature genes in D425 cell lines with and without *METTL3* and *METTL14* knockdown

**Supplementary Table 3.** (A) WGCNA module numbers and colors containing at least one m6A regulator gene. (B) List of co-expressing lncRNA genes within the modules representing at least one m6A regulator gene

**Supplementary Fig. 1.** (A-B) LASSO-Cox regression analysis to identify minimum features associated with OS

**Supplementary Fig. 2.** AUC plots show XGBoost model-based classification accuracy between individual MB subgroups in independent validation cohorts

## Data Availability

No datasets were generated or analysed during the current study.

## Abbreviations

MB: Medulloblastoma
WNT: WNT signaling-activated
SHH: Sonic hedgehog signaling-activated
Grp3: Group 3
Grp4: Group 4
CNA: Copy number alteractions
scRNA-seq: Single cell RNA sequencing
lncRNA: Long noncoding RNA
circRNA: Circular RNA
ncRNA: Noncoding RNA
m6A: N6-methyladenosine
CRC: colorectal cancer
TAM: Tumor-associated macrophage
GBM: Glioblastoma
PCA: Principal component analysis
WGCNA: Weighted gene co-expression network analysis
M6LSig: m6A-associated lncRNA signature
cph: Cox proportional hazards
ROC: Receiver operating characteristic
LDA: Linear discriminate analysis
SVM: Support vector machine
GB: Gradient boosting
RF: Random Forest
KNN: K-nearest neighbors
DEG: Differentially expressed genes
GSEA: Gene set enrichment analysis
TME: Tumor microenvironment
TPM: Transcripts per million
HR: Hazards ratio
AUC: Area under curve
