# Supplementary figures and images for: Systematic transcriptomic analysis of childhood medulloblastoma identifies N6-methyladenosine-dependent lncRNA signatures associated with molecular subtype, immune cell infiltration, and prognosis
Source: Acta Neuropathol Commun. 2024 Aug 28;12:138. doi: 10.1186/s40478-024-01848-2 (PMC11351195; doi:10.1186/s40478-024-01848-2)

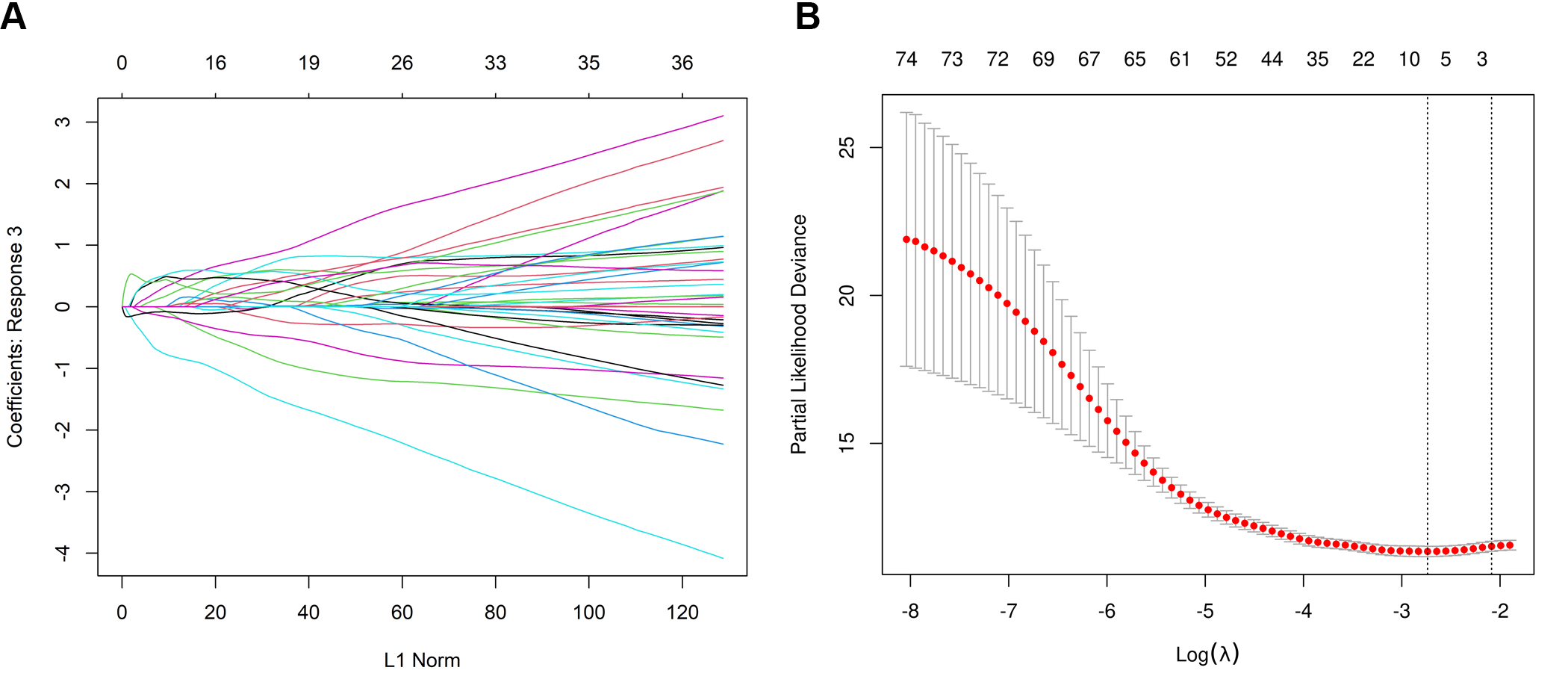

Supplement: Supplementary file 4 — Supplementary Fig. 1. (A-B) LASSO-Cox regression analysis to identify minimum features associated with OS [file 40478_2024_1848_MOESM4_ESM.tif]

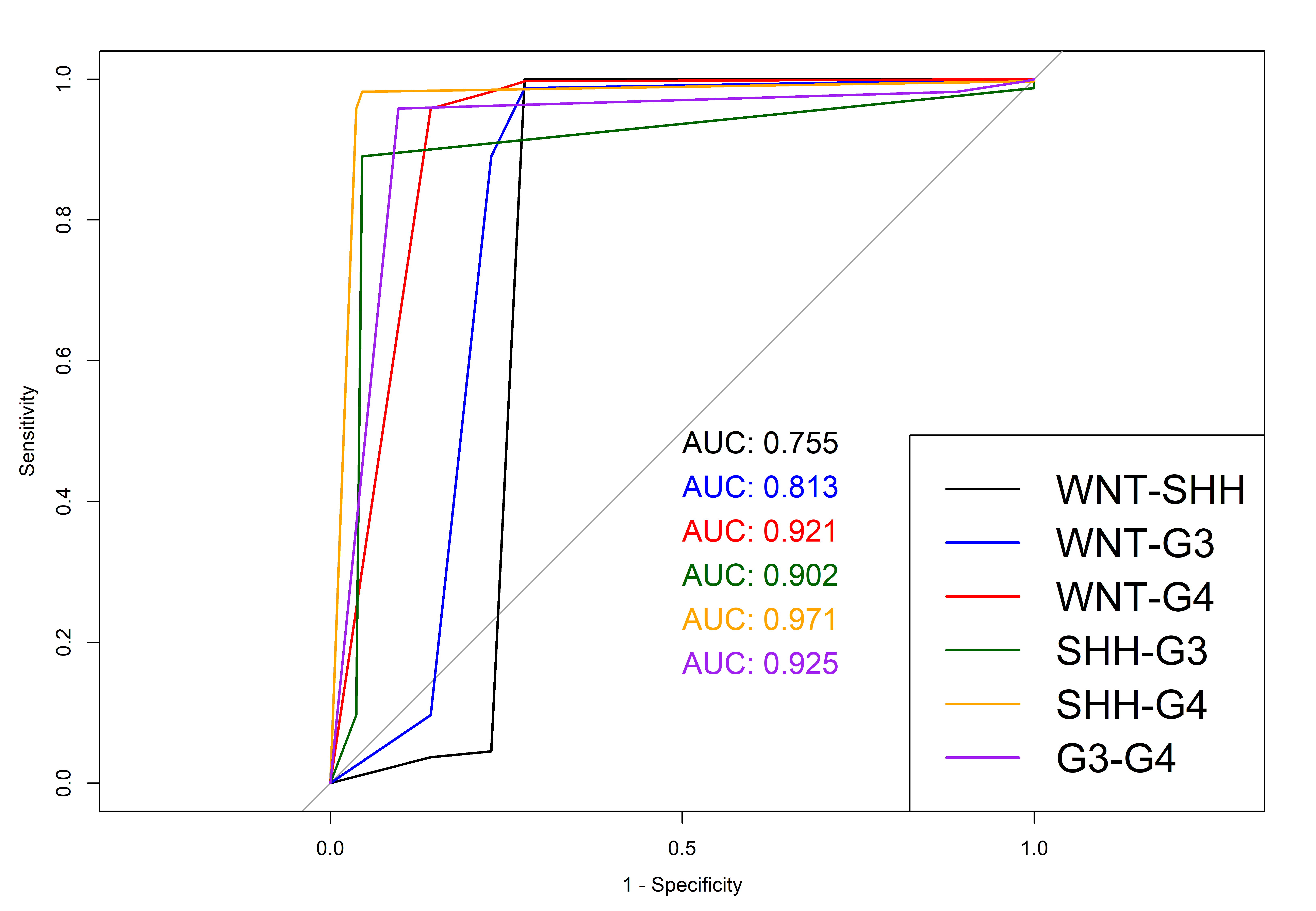

Supplement: Supplementary file 5 — Supplementary Fig. 2. AUC plots show XGBoost model-based classification accuracy between individual MB subgroups in independent validation cohorts [file 40478_2024_1848_MOESM5_ESM.tif]
